# Reduction of Malnutrition Related to Unsafe Water Consumption in Developing Countries: Potabilization of Surface Water and Traditional Well Water, with Plant Extracts

**DOI:** 10.3390/ijerph21050519

**Published:** 2024-04-23

**Authors:** Frédéric Anderson Konkobo, Mamounata Diao, Paul Windinpsidi Savadogo, Roger Dakuyo, Noëlle Edwige Roamba, Sandrine Zongo, Mamoudou Hama Dicko

**Affiliations:** 1 Laboratory of Biochemistry, Biotechnology, Food Technology and Nutrition (LABIOTAN), Department of Biochemistry and Microbiology, University Joseph KI-ZERBO, Ouagadougou 03 BP 7021, Burkina Faso; 2 Soil-Water-Plant Laboratory, Institute of Environment and Agricultural Research, National Center for Scientific and Technological Research (INERA/CNRST), Ouagadougou 01 BP 476, Burkina Faso; 3 International Joint Research Unit—Environment, Health and Societies (UMI 3189, ESS), Ouagadougou 01 BP 476, Burkina Faso

**Keywords:** water, biocoagulant, bioflocculant, turbidity, potabilization

## Abstract

The consumption of unsafe water in rural areas is a real public health problem in developing countries. This situation mainly affects children under five years of age and causes several deaths and many cases of malnutrition every year. The objective of this study was to evaluate and optimize the capacity of four local plant extracts in the potabilization of unsafe water. Thus, *Moringa oleifera* and *Boscia senegalensis* seeds, or *Aloe vera* and *Opuntia ficus-indica* mucilages were prepared in a solution and applied during a jar test as biocoagulants and bioflocculants on three raw water samples of 82.3 NTU, 549.8 NTU and 796.9 NTU. After treatment results showed that 0.9 g/L of Moringa biocoagulant or 1 g/L of Boscia biocoagulant applied with 0.4 mL of *Aloe vera* bioflocculant or 0.6 mL of *Opuntia ficus-indica* bioflocculant reduced the turbidity of each water sample to values less than 5 NTU after only 15 min of decanting. Moreover, the sanitary quality of the water treated by these different extracts showed a perfect conformity of the physicochemical and microbiological parameters with the standards of acceptability in drinking water decreed by the World Health Organization. Thus, the application of these local plant extracts has made it possible to considerably improve the quality of unsafe water in record time. Their popularization could be an alternative in the fight against malnutrition related to the consumption of unsafe water, especially in rural areas.

## 1. Introduction

Drinking water is a fundamental and indispensable resource for the existence of living beings [1]. Considered as a food in its own right, it is essential for good nutrition and the proper functioning of the human body. For this, it must not only be accessible in a sufficient quantity, but above all be of impeccable quality so that it can be consumed without risk to the health of populations [2,3]. 

The sanitary safety and quality of the water consumed are thus essential to human development and well-being. That is why the UN General Assembly adopted a resolution in 2010, in which it declared that the right to drinking water is a “fundamental right, essential to the full exercise of the right to life and all human rights” [4,5]. 

Unfortunately, despite the progress made in the past decades around the world, access to safe drinking water remains one of the major challenges of the 21st century [6]. According to the World Health Organization and United Nations Children Fund (WHO/UNICEF) joint program report (2021), 25% of the world’s population still lacks access to safe water as a source of food [7,8]. Thus, despite the fact that access to safe water is recognized as a human right, nearly 2.2 billion people worldwide still lack access to its services [9].

Worse, in sub-Saharan Africa, the number of people consuming unsafe water on a daily basis increased by 45% between 2000 and 2019. As a result, 2.6 million people die each year from diseases related to unsafe water consumption, making it one of the leading causes of death in the world [10]. That is why water which is supposed to be a source of life, is unfortunately depending on its quality, can be a source of disease and death.

Indeed, if in the majority of developed countries, access to drinking water has practically no problem, it remains a real challenge for developing countries (DC) because it represents a serious handicap in achieving global food security [11]. This problem is much more acute in rural areas where certain disadvantaged populations consume water directly from rivers, swamps and traditional wells without any prior treatment [12,13]. 

Unfortunately, this consumption of unsafe water leads to an increased vulnerability of these populations to waterborne diseases such as diarrhea, cholera, dysentery, typhoid, etc. [14,15,16,17]. All these pathologies are factors that promote malnutrition in populations because they lead to the poor absorption of nutrients by the human body. Thus, the lack of access to drinking water is a real public health problem because of waterborne diseases it causes are closely linked to malnutrition, which can lead to death [18,19,20,21].

According to the World Health Organization (WHO), 50% of child malnutrition in developing countries is due to unsafe water consumption. As a result, 297,000 children under the age of five die each year in these countries from diarrheal diseases caused by unsafe water consumption [3,22,23,24,25]. For many other children who survive, this unfortunately leads to serious health problems, such as undernutrition and stunting, that affect them into adulthood [26,27,28].

With regard to this situation, and in the absence of adequate infrastructure, initiatives to promote individual or domestic water treatment techniques could be a challenge. It is in this context that the WHO recommends simple domestic treatment methods such as boiling, filtration or decantation, which can improve water quality, although to different degrees [29,30]. However, these different techniques have limitations in terms of the mastery of treatment procedures, especially their efficiency [31]. 

The potabilization of unhealthy water by plant extracts is indeed a heritage of the peoples of Asia and Africa. However, with modernization and the invasion of synthetic chemical coagulants, this traditional process has practically disappeared over the years in many countries that have experienced development. 

Nowadays, the exploration of this once-traditional method by a new approach could be a sustainable alternative to reduce malnutrition caused by unsafe water consumption. Unfortunately, most studies on the use of plant extracts in water purification have focused on *Moringa oleifera* seeds as a biocoagulant and their capacity to clarify raw water. Few studies have focused on the optimization of the purification efficiency of these seeds as well as on the efficiency of other local plant extracts found in rural areas of developing countries. Therefore, this study highlights a potabilization technology based on the principle of coagulation/flocculation using local plant extracts. The innovation of this study lies in the fact that it provides new knowledge on the optimal conditions of the use of *Moringa oleifera* seeds, but also of *Boscia senegalensis* seeds as a biocoagulant, and mucilaginous extracts of *Aloe vera* and *Opuntia ficus-indica* as bioflocculants. This study therefore develops an optimized potabilization method based on local plant extracts whose interest lies in the speed of the treatment process, the non-toxicity of the extracts and their accessibility for populations living in rural areas. Thus, the application of this process at the individual and family level should be an alternative for the potabilization of water in developing countries (DC), especially in rural areas. 

## 2. Material and Methods

### 2.1. Collection of Raw Water Samples

The raw water samples used for this study were essentially of two types: surface water and unhealthy groundwater. The surface water was collected at two sites: Loumbila dam and dam n°3 of Ouagadougou.

The Loumbila dam (12°29′ N, 01°24′ W) is located in central Burkina Faso, east of the capital Ouagadougou. It has a capacity of about 42 million m^3^ and an average depth of 2.15 m. 

The, Ouagadougou dam N°3 (01°33′20″ W 12°23′04″ N) is also intended to supply drinking water. It has a capacity of 5.6 million m^3^ and a depth of 2 m. 

Groundwater samples were mainly obtained from an abandoned traditional well in the village of Nasso, a rural commune located a short distance from the city of Bobo Dioulasso (11°12′49″ North, 4°26′12″ West).

All samples at each site were collected in 20 L plastic containers, transported to the laboratory and then stored in a refrigerator at 4 °C in accordance with the French standard NF EN ISO 5667-3 (2004). These collected water samples were of poor organoleptic quality and the main in situ physicochemical characteristics are presented in Table 1.

### 2.2. Collection of Plant Material

The plant extracts whose purifying capacities were highlighted in this study are as follows: *Moringa oleifera* seeds, *Boscia senegalensis* seeds, *Opuntia ficus-indica* mucilage and *Aloe vera* mucilage. *Moringa oleifera* seeds were collected by the National Center of Forest Seeds of Burkina Faso (NCFS). *Boscia senegalensis* seeds were collected in the urban park “bangr weeogo” in Ouagadougou. The stems of *Aloe vera* and *Opuntia ficus indica* were collected from plants in a public garden in the city of Ouagadougou.

These different plant extracts were immediately transported to the laboratory, coded (Table 2) and then preserved before their preparation in the coagulating or flocculating solution.

### 2.3. Preparation of Biocoagulant Solutions

#### 2.3.1. Preparation of Solutions Based on Moringa and Boscia Seeds

The Moringa coagulant (Mo) solution was prepared from mature *Moringa oleifera* seeds obtained from the National Forest Seed Center. These seeds were dehulled and then ground using the technique described by Folkard and Sutherland (2002) [32]. The resulting powder was sieved and 100 g was then dissolved in 1 L of distilled water. The mixture was stirred for 2 h to extract the active compounds, then filtered and stored at 4 °C in the refrigerator according to the method described by kaboré et al. (2018) [33]. If not used directly, the cold storage of the biocoagulant solution thus obtained prevented the alteration of the active substances. A stock solution of Moringa biocoagulant with a concentration of 100 g/L was then obtained. 

This same protocol was applied for the preparation of the solution based on *Boscia senegalensis* seeds. Thus, a stock solution based on Bs extract was obtained and stored under the same conditions as the Mo solution, at a temperature of 4 °C.

#### 2.3.2. Preparation of Mucilaginous Solutions of Cactus and Aloe

The preparations of the mucilaginous extracts of *Opuntia ficus-indica* (Ofi) and *Aloe vera* (Av) were completed separately but underwent the same process that was performed in several steps. To do so, young and healthy stems of each of these plants were properly washed and then cut into small pieces to facilitate grinding. Next, 50 g were then ground with a blender, and the resulting grind was filtered through a sieve and collected in a jar in which it was diluted with 1 L of distilled water and homogenized. Two stock solutions of cactus extract and Aloe were obtained with a concentration of 50 g/L each. It is a slightly viscous liquid with a green color and a pH equal to 3.5. These solutions are relatively stable and can maintain their flocculation capacity for several days outside of any preservation system. For traceability purposes they were stored in the laboratory at 4 °C.

### 2.4. Jar Tests

The evaluation and the optimization of the efficiency of the coagulating and flocculating solutions previously prepared were carried out by jar tests on the collected water samples. The jar tests are indeed small-scale simulations of raw water treatment by coagulation/flocculation. They were carried out during this study with an electrically controlled flocculator with six stations (FC6S Velp Scientifica Jar Test).

To do this, increasing volumes of a biocoagulant (Moringa or Boscia extract) were first introduced into each flocculator beaker, each contained 1 L of the raw water sample to be treated. The flocculator was set under strong agitation at 150 rpm for 5 min. This rapid phase corresponding to the coagulation step consisted of dispersing the biocoagulant everywhere in the water sample in a fast and uniform way. 

Thereafter, the agitation was moderated a second time at 45 rpm for 10 min, preceded by the addition or not of a bioflocculant (Mucilage of cactus or Aloe). This slow phase corresponding to the flocculation stage allowed the agglomeration of small particles to form larger flakes that can settle much faster. The different tests were performed in triplicate for each biocoagulant or bioflocculant concentration tested. These different concentrations were then calculated using the following formula (Equation (1)):

(1) $$C=\frac{C1\times V1}{V}$$

where:

C: Concentration of biocoagulant in the treated water sample (g/L)

C1: Concentration of the prepared biocoagulant stock solution (g/L)

V1: Volume of biocoagulant taken for treatment (L)

V: Total volume of the water sample (L)

The jar tests have thus allowed, during this study, the estimation of the optimal concentrations of the biocoagulant and bioflocculant, as well as the optimal settling time according to the characteristics of the treated water. 

### 2.5. Mechanism Involved during the Jar Test: Coagulation–Flocculation

**Figure 1:**
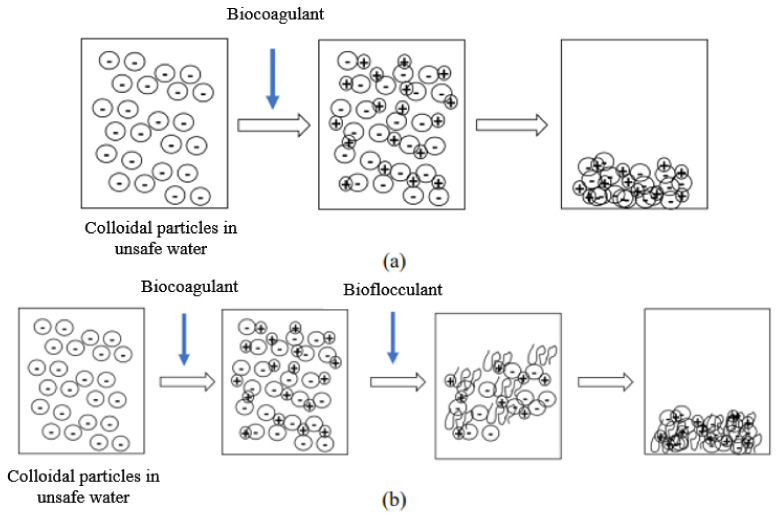
Mechanism of action of the biocogulant during the coagulation–flocculation process (**a**). Mechanism of action of the biocogulant/bioflocculant complex during the coagulation–flocculation process (**b**).

The suspended particles contained in raw water, and which are responsible for its turbidity, are mostly colloids. Unfortunately, these colloids cannot settle naturally because of their small size and because of the repulsion forces that exist between them. Indeed, the surface of these particles is negatively charged, which allows them to repel each other and prevents them from forming larger masses, called flocs; they can therefore not settle. In order to obtain an efficient and faster settling, coagulation and flocculation processes are used. Coagulation is the destabilization of these colloids by neutralizing the forces that keep them separate, thanks to the addition of a coagulant. Flocculation is the agglomeration of these “discharged” particles into larger flocs that can be easily settled (Figure 1).

The particularity of this study is that the coagulants and flocculants used in the potabilization process were plant-based. The coagulants were extracts of *Moringa oleifera* and *Boscia senegalensis* seeds. The flocculants were mucilaginous extracts of *Opuntia ficus-indica* and *Aloe vera*.

### 2.6. Determination of the Quality Parameters of Water Treated with Plant Extracts

#### 2.6.1. Determination of Physicochemical Parameters

The physicochemical parameters measured took into account the main parameters of sanitary impact and acceptability of drinking water. These included turbidity, pH, conductivity, temperature, alkalimetric title (AT), complete alkalimetric title (CAT), hydrometric title (HT), calcium and magnesium hardness (TCa^2+^, TMg^2+^) as well as the content of certain minerals such as sodium and potassium. Turbidity was measured using the nephelometric method by a WTW Turb 550 IR turbidimeter in accordance with the French standard NF ISO 7027 (2000). The results were expressed in nephelometric turbidity units (NTU).

The pH was measured by the electrochemical method using a pH meter (330i WTW) equipped with a combined electrode in accordance with the NF 10523 (1994) method. The pH was expressed in pH units.

Conductivity and temperature were measured using a conductivity meter coupled to a WTW thermometer. The results were expressed in degrees Celsius (°C) for temperature and in micro siemens per cm (μS/cm) for conductivity.

The AT and CAT were determined titrimetrically by dosing 100 mL of the water sample with 0.1 N HCl in the presence of phenolphthalein as a colored indicator for AT, and methyl orange for CAT, in accordance with French standards NF T 9963: 1996. They were expressed in meq/L the relation AT or CAT (in meq/L) = VHCl × 0.02.

The concentrations of calcium, magnesium and total hardness were determined by titrimetric method according to the French standards, NF T 90-016: 1984 for calcium and magnesium, and NF T 90-003: 1984 for total hardness.

Sodium and potassium ions were determined using a flame photometer. Their determination in the water samples consisted of introducing in a beaker a small quantity of the sample in which the probe of the apparatus was introduced, which after a few moments revealed the contents of these ions in mg/L. It should be noted that before any measurement of the content of these ions, the apparatus had been previously calibrated.

#### 2.6.2. Determination of the Microbiological Quality

The bacteriological analysis of the water samples before and after treatment with bio-extracts consisted of looking for the germs that it could contain. For each water sample (raw or treated), the microbiological analysis concerned:

▪ identification and enumeration of total coliforms;
▪ identification and enumeration of enterococci;
▪ identification and enumeration of *Escherichia coli*.

The identification and the enumeration of bacteria in water were carried out by the membrane filtration method according to the French standard NF EN ISO 9308-1 (2000). It consisted of passing 100 mL of water to be analyzed through a membrane of 0.45 µm of size. The membrane, having retained any bacteria present in the water, was placed on an appropriate agar culture medium in a Petri dish. These samples were then incubated for 24 h at different temperatures depending on the germs being tested: 44 °C for enterococci, and 37 °C for *Escherichia coli* and total coliforms. After incubation, the colonies of each group of bacteria were counted and expressed in CFU/100 mL.

Each microbiological parameter was thus determined in triplicate, and the percentage of reduction of microorganisms found (% m) after the application of each biocoagulant in the treatment was determined by the following formula (Equation (2)):

(2) $$\%\;m=\frac{\mathrm{mi}-\;\mathrm{mf}\;}{\mathrm{mi}}\times 100$$

where:

mi: number of colonies of the microorganism initially present in the water (CFU/100 mL.);

mf: number of colonies of the same microorganism after treatment (CFU/100 mL).

### 2.7. Statistical Analysis

Graphs and tables were performed with GraphPad Prism version 8.4.3 and Microsoft Excel version 2016. Data were subjected to analyses of variance (ANOVA) and significant differences between means were revealed by Tukey’s test (*p* < 0.05) and were performed with XLSTAT software (version 2016). A principal component analysis and hierarchical clustering were performed with R software, version 4.0.2 (2020).

## 3. Results and Discussion

### 3.1. Results

#### 3.1.1. Optimal Concentration of *Moringa* Biocoagulant

**Figure 2:**
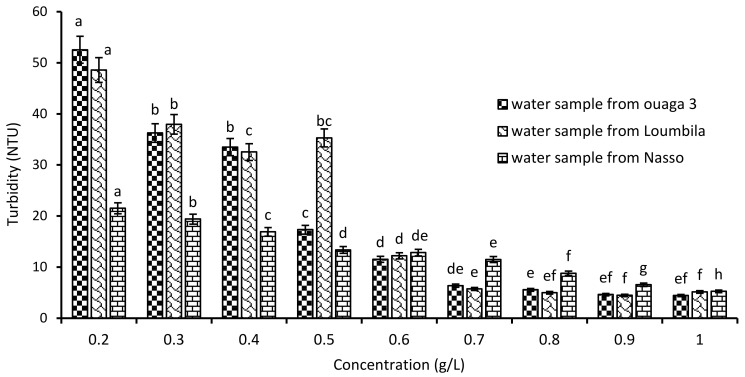
Variation of turbidity of water samples as a function of Moringa Oleifera seeds’ extract concentration. The letters “a, b, c, d, e, f, g, h” indicate the different variations. Sticks with the same letters have statistically not different values. However, sticks with different letters have statistically different values.

Water samples from Ouaga 3 (549.8 NTU), Loumbila (796.9 NTU) and Nasso (82.3 NTU) were used for coagulation/flocculation tests with increasing doses of *Moringa oleifera* seed extract. The variations in residual turbidity for each sample after 2 h of settling are recorded in Figure 2. A turbidity abatement depending on the applied Mo extract concentration was observed. Indeed, as the extract concentration increased, the turbidity of the water decreased until it reached the standard (≤5 NTU) after 2 h of decantation. Thus, for the three water samples, the optimal Mo extract concentration was 0.9 g/L for Ouaga 3 and Loumbila waters, and 1 g/L for Nasso groundwater. This makes an abatement of 99% for the Ouaga 3 and Loumbila water samples, and 93.5% for the Nasso sample.

#### 3.1.2. Optimal Concentration of *Boscia Senegalensis* Extract

**Figure 3:**
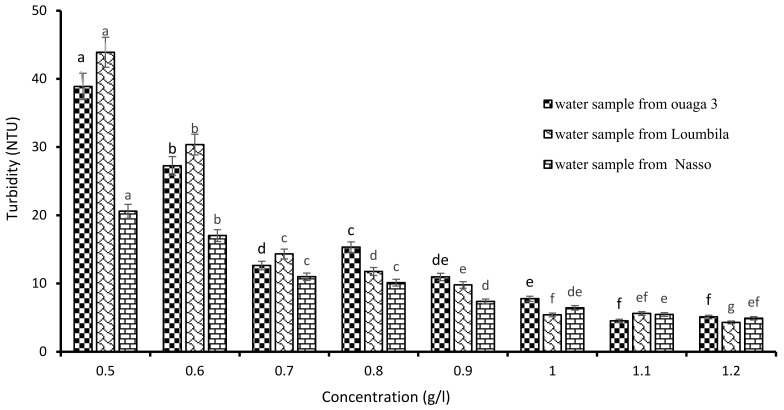
Variation of turbidity of water samples as a function of Boscia senegalensis seeds extract concentration. The letters “a, b, c, d, e, f, g” indicate the different variations. Sticks with the same letters have statistically not different values. However, sticks with different letters have statistically different values.

The determination of the optimal concentration of Boscia extract was carried out by applying increasingly higher concentrations of the latter on the raw water samples (Ouaga 3, Loumbila and Nasso). Figure 3 shows that the turbidity of the water samples decreased as the concentration of Boscia extract increased, until reaching the optimal concentration for each sample after 2 h of decantation. Thus, for the water sample of Ouaga 3, 1.2 g/L of Boscia extract allowed us to reduce the turbidity from 549.8 to 4.9 NTU, that is, with an abatement rate of 99.1%. The same concentration of Boscia extract also reduced the turbidity of the Nasso water sample from 82.3 to 4.3 NTU, i.e., an abatement rate of 94.77%. For the Loumbila water sample, the optimal concentration of Boscia extract was 1.1 g/L and the turbidity removal rate was 99.43%.

#### 3.1.3. Optimization of the Efficiency of Mo and Bs Biocoagulants by Combination with Cactus and Aloe Mucilages

The decantation of colloids in dirty water obeys Stokes’ law. This law shows that the falling speed of a particle is proportional to the difference in density between the particle and the liquid, and to the square of the particle diameter. Therefore, any phenomenon that increases the diameter of the particles, such as the addition of a flocculating agent, promotes sedimentation. That is why the optimization of the capacity of the biocoagulants Mo and Bs consisted of first applying the seeds of *Moringa oleifera* and *Boscia senegalensis* as a coagulant, then the mucilages of *Aloe vera* and *Opuntia ficus-indica* as the flocculant a second time. Four combinations of the type of biocoagulant/bioflocculant were obtained: Moringa/cactus, Moringa/Aloe, Boscia/cactus and Boscia/Aloe. These combinations were applied on the three collected water samples.

##### Combination of Mo Seeds Extract and Cactus Mucilage

**Figure 4:**
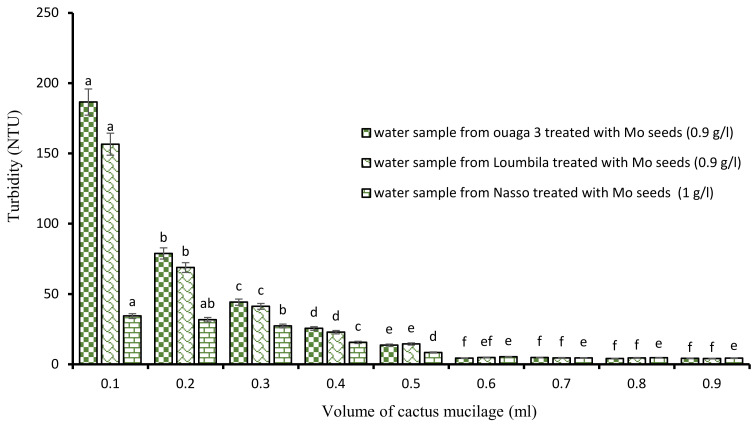
Turbidity variation depending on the volume of cactus mucilage added in Moringa seeds-based treatment. The letters “a, b, c, d, e, f” indicate the different variations. Sticks with the same letters have statistically not different values. However, sticks with different letters have statistically different values.

After the application of the biocoagulants, the minimum settling time to obtain water with turbidity in compliance with the standard (≤5 NTU) was 2 h. To reduce this relatively long time, we proceeded to a combination of the biocoagulant/bioflocculant type by associating Moringa extract and cactus mucilage. To do so, the turbidity of each water sample was monitored by the jar test with the optimal concentrations of Moringa extract combined with increasing volumes of cactus extract (ranging from 0.1 mL to 0.9 mL). These different tests showed that the turbidity abatement was dose-dependent (Figure 4), and that from a certain concentration, 15 min of decantation was sufficient to obtain water with turbidity in compliance with the standard (≤5 NTU). Indeed, as the volume of cactus extract increased, the turbidity of the water decreased. Thus, from 0.6 mL of cactus extract applied to the different optimal concentrations of Moringa, we obtained for each sample of treated water a clear water of compliant turbidity. 

##### Combination of Bs Seeds Extracts and Cactus Mucilage

**Figure 5:**
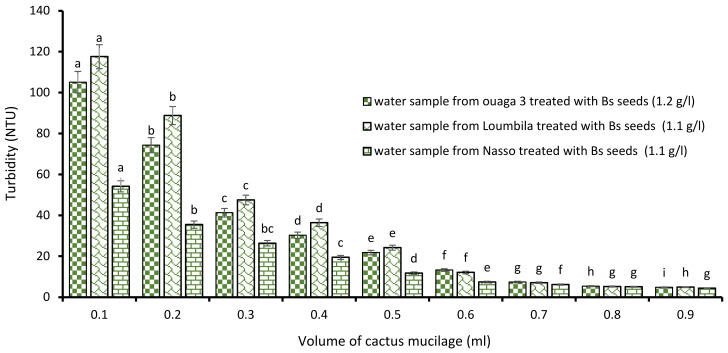
Turbidity variation depending on the volume of cactus mucilage added in Boscia seeds-based treatment. The letters “a, b, c, d, e, f, g, h, i” indicate the different variations. Sticks with the same letters have statistically not different values. However, sticks with different letters have statistically different values.

The combination “Boscia extract/cactus mucilage” also allowed to obtain a compliant turbidity after 15 min of decantation for each raw water sample. Indeed, this new combination, as with the first one, was dose-dependent, and more the volume of cactus extract applied to the different optimal concentrations of Boscia extract increased, more the turbidity of the water considerably decreased. Thus, 0.9 mL of cactus extract associated with each optimal concentration of Boscia extract for the treatment of the Ouaga 3, Loumbila and Nasso catchments reduced their turbidity to less than 5 NTU after only 15 min of decantation (Figure 5).

##### Combination of Mo Seeds *Aloe vera* Mucilage

**Figure 6:**
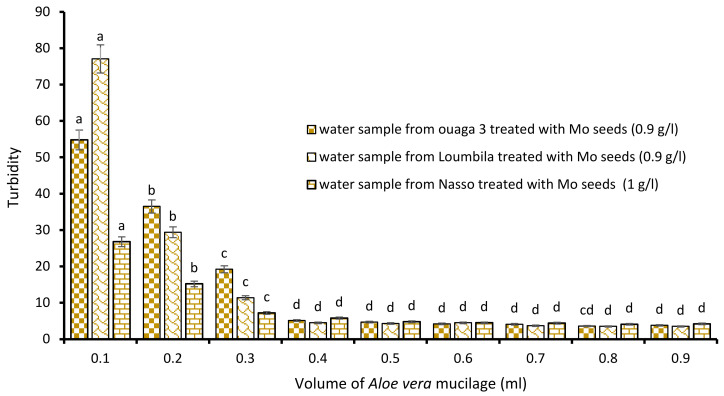
Turbidity variation depending on the volume of Aloe mucilage added in Moringa seeds-based treatment. The letters “a, b, c, d” indicate the different variations. Sticks with the same letters have statistically not different values. However, sticks with different letters have statistically different values.

A new combination of the biocoagulant/bioflocculant type has made it possible to considerably reduce the settling time of the water once the bioextracts have been applied: this is the Moringa/*Aloe vera* combination (Figure 6). The application of increasing volumes of *Aloe vera* at the optimal concentration of Moringa allowed it to reach turbidities lower than 5 NTU for each sample after 15 min of decantation. Thus, for the raw water sample from Ouaga 3, the successive addition of 0.9 g/L of Moringa extract during the coagulation step, and 0.4 mL of Aloe sap during flocculation reduced the turbidity of the sample from 549.8 to 4.7 NTU after only 15 min of decantation. The same was true for the Nasso water sample, where the combination of 0.9 g/L Moringa extract and 0.4 mL Aloe sap reduced the turbidity of the sample from 82.3 to 4.5 NTU. For the raw water from Loumbila (796.9 NTU), only 0.4 mL of Aloe mucilage was required to obtain a final turbidity of 4.5 NTU with the optimal Moringa concentration. 

##### Combination Bs Seeds/*Aloe vera* Mucilage

**Figure 7:**
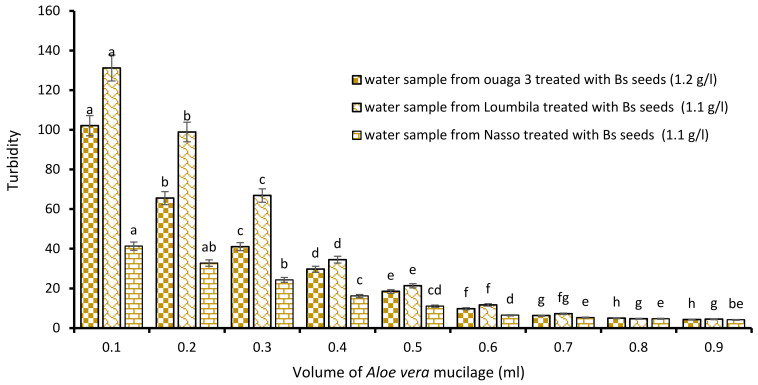
Turbidity variation depending on the volume of Aloe mucilage added in Boscia seeds-based treatment. The letters “a, b, c, d, e, f, g, h” indicate the different variations. Sticks with the same letters have statistically not different values. However, sticks with different letters have statistically different values.

The last biocoagulant/bioflocculant combination consisted of Boscia extract and *Aloe vera* extract. The addition of increasing volumes from 0.1 to 0.9 mL of *Aloe vera* extract to the Boscia extract allowed it to reduce considerably the turbidity of the raw water samples. The level of water turbidity after 15 min of decantation was dependent on added concentrations of *Aloe vera* extract to the optimal concentrations of Boscia (Figure 7). For all three samples (Ouaga 3, Loumbila and Nasso), the optimal volume of Aloe mucilage was 0.8 mL. This volume applied to the Boscia concentrations for each treatment, resulted in a turbidity of 4.4 NTU, 4.9 NTU and 4.9 NTU for the Ouaga 3, Loumbila and Nasso samples, respectively.

#### 3.1.4. Comparison of the Efficiency of the Different Combinations

First, the application of biocoagulants only (Moringa and Boscia seeds) allowed a good reduction of turbidity, but the settling time was long (2 h). In order to reduce this time and improve the efficiency of the process, the combination of cactus and Aloe mucilage as bioflocculants was carried out. The four biocoagulant/bioflocculant combinations allowed us to considerably improve the turbidity removal rate of the raw water samples in a record time of 15 min. These combinations allowed for each water sample to reduce turbidity to almost 99% after only 15 min of decantation.

**Figure 8:**
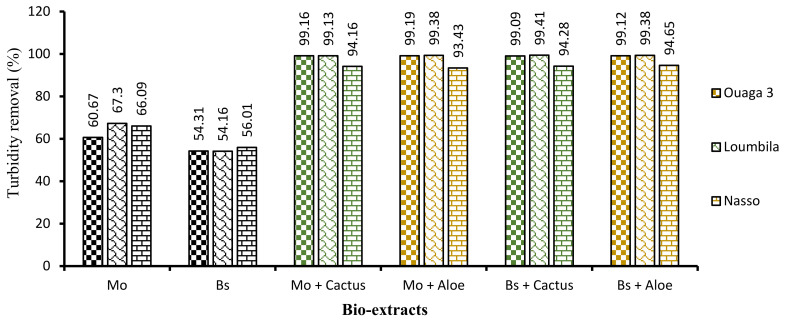
Turbidity abatement of water samples as a function of bioextracts applied after 15 min of decantation.

**Figure 9:**
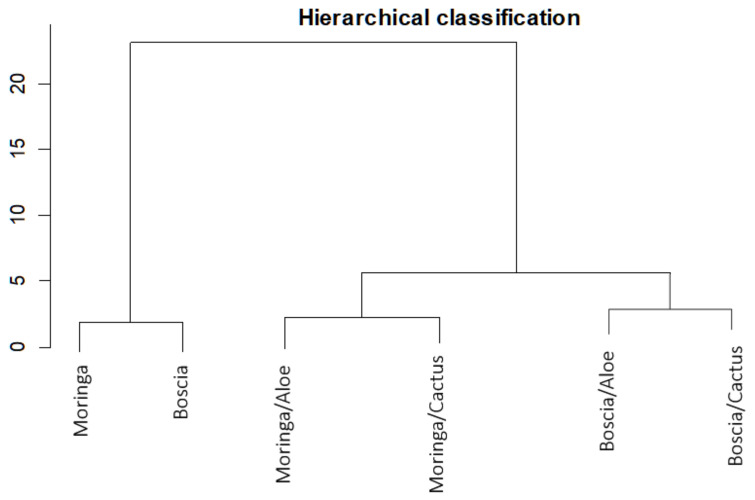
Hierarchical classification of the performance of biocoagulants and bioflocculants in the removal of turbidity from unsafe water samples.

Analyzing Figure 8, which shows the proportion of turbidity removal for each extract and for each combination, we can deduce in addition to the hierarchical classification (Figure 9) that Moringa seeds were more effective than Boscia seeds. As for the flocculants, Aloe mucilage seemed to be more effective than cactus mucilage because the optimal volumes of Aloe mucilage remained lower than those of cactus mucilage for the treatment of the same water samples and under the same conditions. We can therefore deduce that the Moringa/Aloe complex was the best combination, followed successively by Moringa/cactus, Boscia/Aloe and Boscia/cactus complexes.

#### 3.1.5. Effects of Biotreatments on Physicochemical Parameters of Water Samples

The determination of physicochemical parameters was performed in triplicate and the obtained means are recorded in Table 3. The analysis of variances at *p* > 0.05 showed that the biotreatments applied had a significant effect on almost all physicochemical parameters of the water samples, except for pH and AT. Indeed, the addition of Moringa, Boscia, Aloe and cactus extracts did not lead to a significant variation of the pH whose value remained in the interval [6.8–7.8]. Additionally, the Alkalimetric Title (AT) for each water sample before the application of the bioextracts was 0°F and did not vary during the different treatments.

For other parameters such as CAT, HT, conductivity, as well as calcium, magnesium, sodium and potassium contents, the variations were significant compared to the raw water samples, whatever the biocoagulant or bioflocculant applied during the treatments.

Thus, we note that the Complete Alkalimetric Title (CAT) decreased after application of the different bioextracts. On the other hand, the total hardness (HT), taking into account the contents of calcium and magnesium ions, generally increased slightly in the waters after each biotreatment. The same was true for the conductivity, sodium and potassium content.

**Figure 10:**
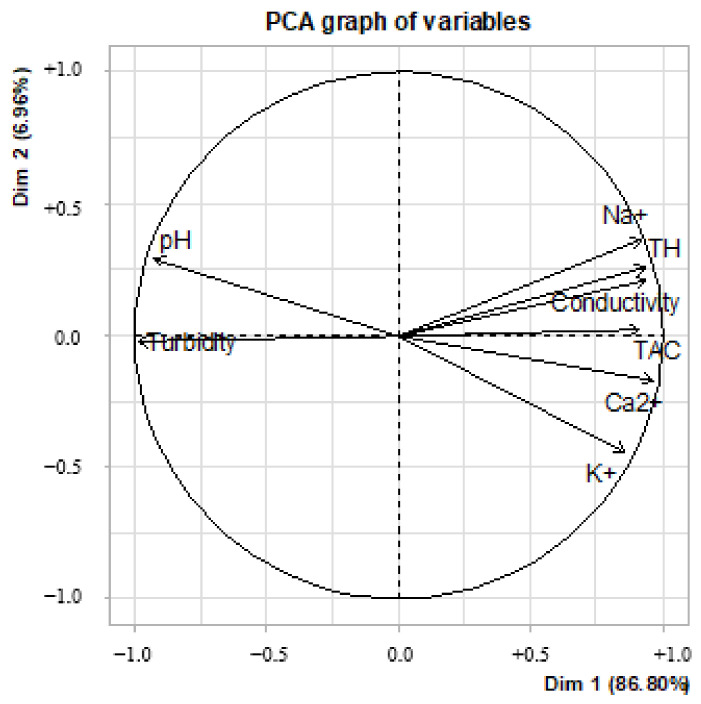
Principal component analysis of physicochemical parameters of water treated with plant extracts.

The results of the principal component analysis (Figure 10) allow us to observe two groupings: Dim 1 (86.8%) and Dim 2 (6.96%). It can be seen that on the Dim 1 axis, sodium, potassium, calcium, HT, CAT and conductivity were grouped together. Indeed, in this grouping, the association of these ions corresponded to an increase in mineralization in the treated water samples. This increase was translated here by the contribution of cations by the plant extracts used during the various treatments.

**Figure 11:**
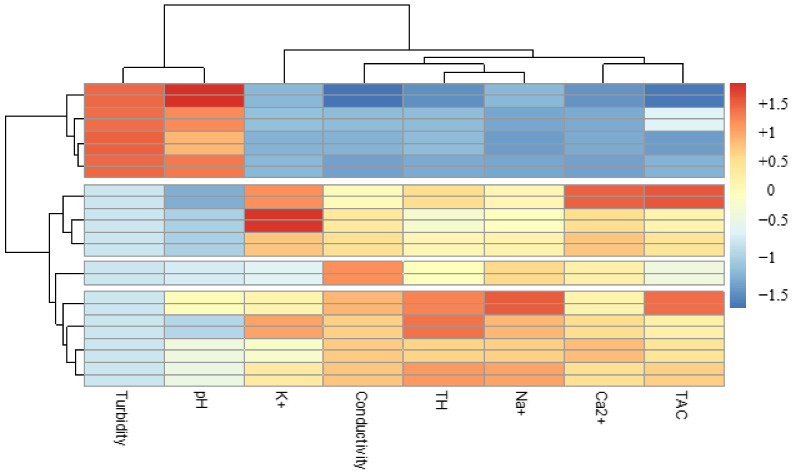
Classification of physicochemical parameters of waters treated with bioextracts.

The second grouping, although not very representative (6.96%), took into account turbidity and pH. These two parameters showed a certain constancy in the waters treated by the different associations with bioextracts (Figure 11). Thus, whatever the biocoagulant or bioflocculant used, they managed to reduce the turbidity of the water to values close to 05 NTU, without major modification of the pH.

#### 3.1.6. Effects of Biotreatment on Microbiological Parameters

**Figure 12:**
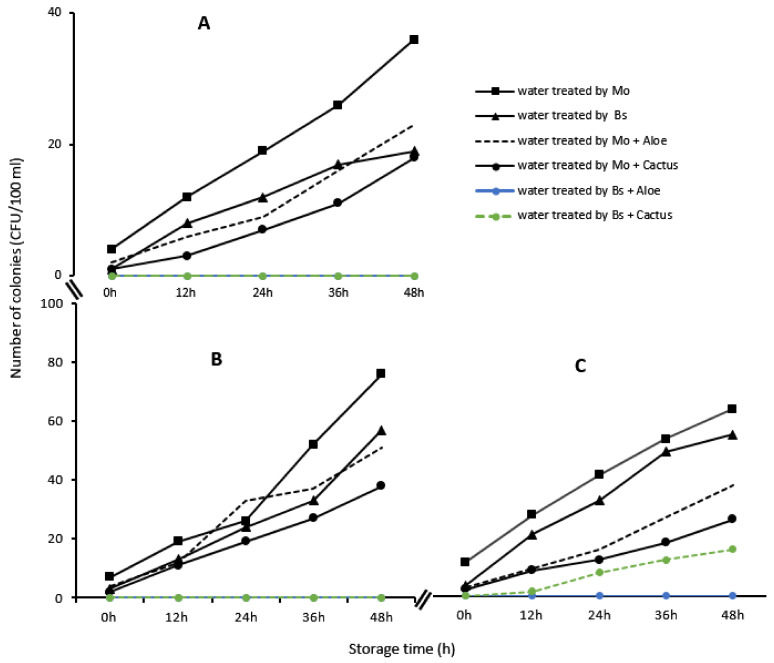
Evolution of *Escherichia coli* (**A**), total coliforms (**B**) and enterococci (**C**), in the samples of water treated from Loumbila, Ouaga 3 and Nasso, respectively, by the bioextracts as a function of conservation time.

The microbiological analyses showed a considerable reduction in the different microbial indicators in each water sample treated with bioextracts. Thus, *Escherichia coli*, total coliforms and enterococci, initially present in the raw water samples, underwent a reduction of almost 99% after the treatment with Moringa seeds, Boscia as well as with the different combinations with cactus sap and *Aloe vera* (Table 4). However, the abatement of microorganisms was not total at times (100%), which implies that some microorganisms remained during the treatment. Thus, it was found that these proliferated again in the treated water during storage, especially if its use was delayed (Figure 12).

To counteract this proliferation, each biotreatment was combined with the process of boiling the treated water in order to definitively destroy any remaining pathogenic germ. After decantation of the waters treated with *Moringa oleifera* seeds, *Boscia senegalensis* and different combinations with *Aloe vera* and cactus sap, these waters were heated to boiling. After boiling, the treated waters were cooled to room temperature and the research of the different microbial indicators such as *Escherichia coli*, total coliforms and enterococci showed a total absence of microorganisms in the water samples.

### 3.2. Discussion

Most biocoagulants and bioflocculants based on plant extracts work primarily through an interparticle bridging adsorption and coagulation mechanism [34,35]. The results of this study also support the hypothesis of an adsorption facilitated by an interparticle bridging mechanism, where the destabilization of the particles takes place thanks to the active principles of each bioextract [36]. 

In fact, the coagulation or flocculation capacity of the plant extracts studied comes from the presence of functional groups and charges incorporated in their structure. Thanks to these compounds, the bioextracts were able to neutralize the colloidal loads that exist in unsanitary waters and to gather these polluting particles into flocs that can settle much more easily [37,38]. Usually, the active principles of biocoagulants are oligosaccharides, lipids or proteins, while those of bioflocculants are mainly polysaccharides and amino acids [35,37,38]. 

It is in this sense that studies have revealed the capacity of *Moringa oleifera* seeds for the treatment of raw water as a biocoagulant [39,40,41,42,43]. It has been proven that Moringa oleifera seeds are among the best plant coagulants discovered to date, whose effectiveness is comparable to certain mineral coagulants, such as aluminum sulfate [40,41]. This purification capacity allows them to improve the quality of unhealthy water and make it drinkable. This property of *Moringa oleifera* seeds is due to their richness in active cationic polyelectrolytes [44] that neutralize colloidal materials and cause the sedimentation of mineral and organic particles [45]. Indeed, according to Moulin et al. (2012), Moringa seeds contain positively charged proteins that bind to part of the surface of negatively charged colloids through electrostatic interactions [46,47].

In 1995, Gassenschmidt et al. were able to isolate a molecule among many others from *Moringa oleifera* seeds that had flocculent properties [48]. This molecule involved in the coagulation and purification mechanism of raw water is a 60 amino acids containing protein (MO2.1, accession: Q93YG0) [49,50].

The MO2.1 protein, also called MOCP (Moringa oleifera cationic protein) [46,51], is a dimeric protein of 13 kDa with subunit of 6.5 kDa. MO2.1 molecules once in raw water attach to surfaces of negatively charged mineral and organic particles such as silt, clay, bacteria, etc., where they will be removed by adsorption due to electrostatic interactions [51,52]. Due to the collision of particles and neutralization, flocs are produced which are deposited by sedimentation under the effect of gravity, leaving the water more or less clear [53].

However, the particularity of our study concerning the purifying capacities of these seeds was that it highlighted the possibility of considerably reducing the decantation time during the treatment by associating the mucilages of *opuntia ficus-indica* and *Aloe vera* (here considered as bioflocculants). These different combinations of the biocoagulant/bioflocculant type have thus made it possible to obtain, in record time (less than 15 min of decantation), water of impeccable quality that can be consumed without risk to human health. This considerable reduction of the decantation time from 2 h to 15 min further improved the efficiency of *Moringa oleifera* seeds in water treatment.

Another finding of this study was the promotion of *Boscia senegalensis* seeds as a coagulant in water treatment. Indeed, they showed very interesting coagulant capacities similar to those of Moringa seeds in water treatment. According to Jahn (1989b), the active principle of this coagulant would also be cationic polypeptides whose mechanism of action is based on the adsorption and neutralization of colloidal particles present in water. However, the capacity of *Boscia senegalensis* seeds in the potabilization of water was optimized during the study by the mucilages of cactus and Aloe which allowed to obtain, similar to the preceding combinations with the Moringa seeds, drinking water in 15 min of decantation instead of 2 h. In addition, the results of this study on the capacities of the seeds of *Boscia senegalensis* to purify the insalubrious waters also enrich the literature, because there is little information on the activity of this biocoagulant, though it is quite as effective as the seeds of *Moringa oleifera*.

Regarding the mechanism of action of *Opuntia ficus-indica* (OFI) mucilage as a bioflocculant during treatment, the adsorption of colloids previously neutralized during coagulation takes place on the main and side chains of the polysaccharides it contains, forming particle–polysaccharide–particle complexes. Adsorption then occurs through dipole–dipole interactions and hydrogen bonds. Due to the high molecular weight of polysaccharides, their long chains can stretch in the medium and adsorb more destabilized colloids. In addition, the natural electrolytes in mucilage, including the divalent cations Ca^2+^ and Mg^2+^, also have a synergistic effect on the adsorption of pollutants. Thus, the active principle of cactus mucilage is not necessarily a particular compound. All of its constituents, i.e., pectic polysaccharides, other polymers and natural electrolyte components, are responsible for its flocculation ability [54]. Pectic polysaccharides among all these components contribute mainly to this capacityand the efficiency of the process is enhanced by the natural electrolytes present in the mucilage [55].

The mechanism of flocculation with *Aloe vera* mucilage is almost similar to that of OFI. *Aloe vera* mucilage contains glyco-aloe-modinanthrone and tannins, which are flocculation aids and act by adsorption with colloids and suspended matters [56]. Benalia et al. (2021) [57] indicate in turn the presence of amide (-NH) and carboxyl (-COOH) groups, which they believe are responsible for the elimination of microflocs formed during the coagulation of the untreated water. Thus, during the flocculation process with *Aloe vera* mucilage, its functional groups that are the carboxyl and amide groups act as adsorption sites for suspended and colloidal particles [57,58].

The analysis of the physicochemical parameters of the water samples before and after treatment with bioextracts showed that the latter had very little influence on the pH. Indeed, for the different treatments, whether they were based on Moringa seeds, Boscia, cactus or Aloe mucilage, the application of none of these bioextracts seemed to significantly modify the pH of the waters during the treatment. Some researches [59,60] go in the same direction by demonstrating that the application of plant extracts in the treatment of water has no significant effect on the pH. 

It was also noted that there was a general increase in the conductivity of the water after each biotreatment. This increase is explained by the contribution of mineral salts by the bioextracts which will ionize during contact with water [61,62]. Among these metal ions, the increase is more in favor of cations such as Ca^2+^, Mg^2+^, Na^+^ and K^+^. The increase in Ca^2+^ and Mg^2+^ concentrations implies an increase in the hydrotitrimetric title of the water due to the fact that the main biocoagulants, the Moringa and Boscia seeds, are rich in these bivalent cations [51,53]. 

The alkalinity of the water samples before and after treatment was assessed through the determination of the AT and CAT, which are two complementary parameters giving an indication of the content of hydroxide, carbonate and bicarbonate ions. With the values of AT being zero for all samples during this study, it was assumed that the contents of hydroxide ion and carbonate ion were very low or even absent. On the other hand, the CAT which included the bicarbonate ions in addition to the previous ions, was reduced in the waters after the application of each bioextract. This is due to the fact that with bicarbonate ions being anions, the application of the bioextracts will allow the release of cationic proteins (in the case of Moringa) and various cationic polyelectrolytes (in the cases of the other bioextracts) that attach to the surfaces of the negatively charged mineral particles, which will be removed by adsorption as a result of electrostatic interactions [47,52]. 

In the end, during this study, the majority of the physicochemical parameters of the water treated by the different bioextracts were in conformity with the standards recommended by the WHO.

The microbiological analysis of the water treated with the bioextracts also showed very interesting results, especially in the reduction of *Escherichia coli*, total coliforms and enterococci. Thus, in the first step, the application of Moringa and Boscia bioextracts allowed an average reduction of 90% of bacteria for each treated water sample. This significant reduction supported the hypothesis that *Moringa oleifera* and *Boscia senegalensis* seeds’ powder has antimicrobial properties against Gram-negative and Gram-positive bacteria [63,64]. The combination of these two coagulants with cactus and Aloe mucilages allowed an average reduction of 98 to 100% of the different microbial indicators and was explained by the fact that the decanting time was short now.

However, it should be noted that if the microbial abatement was not total in the treated water sample and it was stored for a long time at room temperature, we observed again a proliferation of bacteria. This is due to the fact that the storage time was long, and the organic matter from the various bioextracts and which was found in the water will thus serve for the proliferation of those bacteria having escaped treatment. To this effect, Gupta et al. (2021) [65] showed in their study that treatment with Moringa seeds induces an increase in the concentration of organic matter in treated water, and they explained this is due to the high level of organic matter in this coagulant. Shan et al. (2017) [66], also showed, in the same direction that *Moringa oleifera* seeds contain almost 94% organic matter and that the organic matter leads to an increase in the organic matter level in the treated water. This is why the process of boiling the treated water after decantation has been associated with the definitive destruction of any remaining pathogenic germs. Indeed, the boiling treatment is relatively simple to implement and allows the destruction of the totality of germs and micro-organisms present in water [67]. However, for this, the water must first be decanted, as was the case in our study, before being boiled at 100 °C. In practice, the WHO recommends heating the water to a rolling boil for 1 to 3 min. It may happen that the water obtained after boiling has a bland taste, but this problem can be solved by shaking the water vigorously to re-oxygenate it or by adding a little amount of salt.

## 4. Conclusions

Many scientific studies have been conducted in recent years on the valorization of plant extracts in the treatment of drinking water. Many of them have focused on the capacity of bioextracts to clarify water, and very few have focused on the optimization of their efficiency.

The present study has particularly led to the formulation of biocoagulant/bioflocculant complexes based on plant extracts which allowed considerable improvement in the quality of unsafe waters, and in a record time. Thus, it was demonstrated during the study that the seeds of *Moringa oleifera* and *Boscia senegalensis* were excellent coagulants because they allowed the passage from a cloudy water to a clear water after 2 h of decantation. Moreover, the capacities of these two extracts were further enhanced when combined with the mucilages of *Aloe vera* and *Opuntia ficus-indica*, which in turn acted as excellent flocculants, enabling clear water to be obtained in only 15 min of decantation.

These results are very encouraging, because the evaluation of the sanitary quality of the water treated by these different extracts showed perfect conformity with the standards decreed by the WHO for drinking water. However, future perspectives are to be considered in order to better understand the structure and the mechanism of action of each extract. Thus, additional research such as the determination of the porosity of the different extracts, isothermal models and the realization of a scanning electron microscopy are necessary in order to better identify the structure of the active principles and to better understand the mode of action of each extract. This would contribute to a better valorization of these biocoagulants and bioflocculants and would allow their effective application in the improvement of the quality of unhealthy waters in developing countries.

## Figures and Tables

**Table 1:** Characteristics of the samples of unsafe water collected.

	Raw Water Samples			
Parameters	Loumbila	Ouaga 3	Nasso	Norms in Drinking Water (WHO)
Turbidity (NTU)	796.9	549.8	82.3	<5
Temperature (°C)	24.96	26.5	22.3	-
Conductivity (μS/cm)	82.43	216.33	176.70	<500
pH	6.8	7.5	7.8	6.5–8.5
Color	dirty	dirty	dirty	clear
Odor	bad	bad	bad	good

**Table 2:** Plant extracts used in the treatment of unsafe water during this study.

Plant Species	Harvest Area	Part	Code	Images of the Part
*Moringa oleifera*	NCFS/Ouagadougou	Seeds	Mo	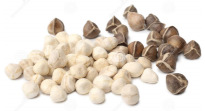
*Boscia senegalensis*	Urban park “Bangr Weeogo” of Ouagadougou	Seeds	Bs	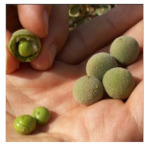
*Opuntia ficus-iondica*	Public garden/Ouagadougou	Mucilage	Ofi	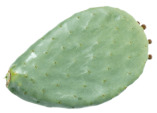
*Aloe vera*	Public garden/Ouagadougou	Mucilage	Av	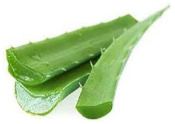

**Table 3:** Variation of physicochemical parameters depending on the treatments applied.

Samples	Treatment	pH	CAT (mg/mL)	HT (mg/mL)	Ca^2+^ (mg/mL)	Mg^2+^ (mg/mL)	Na^+^ (mg/mL)	K^+^ (mg/mL)	Conductivity (μS/cm)
**Ouaga 3**	Raw water	7.5 ^a^	1.06 ^ab^	51.76 ^dee^	33.36 ^bb^	18.40 ^b^	10.93 ^a^	8.000 ^a^	216.33 ^b^
	Ms	7.2 ^a^	1.51 ^aab^	53.53 ^dde^	37.90 ^bb^	15.36 ^bd^	11.76 ^a^	7.733 ^a^	233.96 ^c^
	Bs	7.0 ^a^	1.16 ^ab^	46.23 ^ef^	21.43 ^cc^	24.80 ^ab^	15.83 ^b^	9.933 ^abc^	232.83 ^c^
	Mo + Av	7.7 ^a^	1.81 ^aa^	60.86 ^bcc^	40.60 ^abb^	20.26 ^b^	14.23 ^ab^	13.06 ^a^	208.33 ^a^
	Bs + Av	6.9 ^b^	1.82 ^aa^	56.80 ^cd^	38.60 ^bb^	18.20 ^b^	13.80 ^ab^	11.633 ^aab^	255.06 ^d^
	Mo + Ofi	7.1 ^a^	1.04 ^ab^	65.56 ^bb^	33.53 ^bb^	32.03 ^a^	8.86 ^c^	10.100 ^abc^	253.16 ^d^
	Bs + Ofi	6.9 ^b^	1.32 ^aab^	74.56 ^aa^	51.16 ^aa^	23.40 ^ab^	10.46 ^a^	9.933 ^abc^	233.50 ^c^
**Loumbila**	Raw water	6.8 ^a^	0.68 ^bc^	26.60 ^dd^	14.13 ^cd^	12.46 ^c^	1.95 ^cd^	4.63 ^bd^	82.43 ^abc^
	Ms	6.7 ^a^	0.81 ^bc^	27.96 ^dd^	13.83 ^cd^	14.13 ^b^	6.50 ^ab^	7.93 ^abc^	90.63 ^aab^
	Bs	6.7 ^a^	1.26 ^abb^	35.10 ^cdc^	20.33 ^bcc^	14.76 ^b^	4.13 ^bc^	9.40 ^abc^	91.43 ^aa^
	Mo + Av	6.5 ^a^	0.87 ^bbc^	41.90 ^bcb^	22.40 ^abc^	19.76 ^ab^	4.43 ^bc^	11.16 ^aa^	91.93 ^aa^
	Bs + Av	6.5 ^a^	0.79 ^bc^	47.90 ^bb^	28.63 ^aba^	19.26 ^ab^	7.90 ^aa^	10.53 ^aab^	80.60 ^ac^
	Mo + Ofi	6.6 ^a^	0.95 ^bbc^	45.53 ^bb^	27.80 ^abab^	17.73 ^bbc^	7.73 ^aa^	8.26 ^ac^	85.63 ^abc^
	Bs + Ofi	6.6 ^a^	1.72 ^aa^	60.63 ^aa^	32.23 ^aa^	28.06 ^a^	4.90 ^abc^	8.86 ^abc^	79.70 ^ac^
**Nasso**	Raw water	7.8 ^a^	1.52 ^a^	30.00 ^a^	194.66 ^a^	109.00 ^a^	3.13 ^a^	2.86 ^aa^	176.70 ^bb^
	Ms	7.2 ^ac^	1.16 ^c^	59.93 ^bcd^	32.03 ^bc^	27.90 ^bc^	1.13 ^ac^	2.60 ^aa^	196.00 ^aba^
	Bs	7.0 ^c^	0.94 ^bc^	69.76 ^bc^	38.53 ^bbc^	31.23 ^bc^	2.00 ^a^	2.86 ^aa^	197.23 ^aa^
	Mo + Av	6.9 ^c^	0.93 ^bc^	52.06 ^cd^	29.50 ^bc^	22.56 ^bc^	1.40 ^abc^	2.76 ^aa^	196.63 ^aba^
	Bs + Av	6.8 ^c^	0.98 ^bc^	65.56 ^bc^	31.36 ^bc^	34.20 ^b^	2.33 ^abc^	2.23 ^aba^	190.93 ^aba^
	Mo + Ofi	7.0 ^c^	0.86 ^b^	42.63 ^de^	22.13 ^ce^	20.50 ^c^	2.90 ^b^	1.06 ^bb^	185.00 ^ab^
	Bs + Ofi	6.8 ^c^	0.80 ^b^	72.53 ^b^	41.30 ^b^	31.23 ^bc^	2.86 ^b^	2.40 ^aba^	196.10 ^aba^
Norm	-	6.5–8.5	-	-	-	-	-	-	<500

At the level of each column, the values that have the same letter in common are not significantly different according to the Tukey test.

**Table 4:** Variation of microbiological parameters depending on the treatments applied.

Sample	Treatment	Total Coliforms (UFC/100 mL)	*Escherichia Coli* (UFC/100 mL)	Enterococci (UFC/100 mL)
Ouaga 3	Raw water	313 ^a^	58 ^a^	0 ^a^
	Mo	5 ^b^	1 ^b^	0 ^a^
	Bs	6 ^b^	0 ^b^	0 ^a^
	Mo + Aloe	0 ^c^	1 ^b^	0 ^a^
	Bs + Aloe	0 ^c^	0 ^b^	0 ^a^
	Mo + Cactus	0 ^c^	2 ^b^	0 ^a^
	Bs + Cactus	0 ^c^	0 ^b^	0 ^a^
Loumbila	Raw water	111 ^a^	31 ^a^	4 ^a^
	Mo	14 ^b^	5 ^b^	1 ^b^
	Bs	8 ^bc^	2 ^c^	0 ^b^
	Mo + Aloe	1 ^c^	1 ^c^	0 ^b^
	Bs + Aloe	0 ^c^	0 ^c^	0 ^b^
	Mo + Cactus	0 ^c^	0 ^c^	0 ^b^
	Bs + Cactus	0 ^c^	0 ^c^	0 ^a^
Nasso	Raw water	532 ^a^	97 ^a^	0 ^a^
	Mo	8 ^b^	5 ^c^	0 ^a^
	Bs	3 ^b^	16 ^b^	0 ^a^
	Mo + Aloe	3 ^b^	1 ^c^	0 ^a^
	Bs + Aloe	2 ^b^	1 ^c^	0 ^a^
	Mo + Cactus	1 ^b^	0 ^c^	0 ^a^
	Bs + Cactus	2 ^b^	0 ^c^	0 ^a^
Norm	-	0	0	0

At the level of each line, values that have the same letter in common are not significantly different according to the Tukey test.

## Data Availability

The data and materials used during the current study are available from the corresponding author on reasonable request.
